# Inflammation, Microcalcification, and Increased Expression of Osteopontin Are Histological Hallmarks of Plaque Vulnerability in Patients with Advanced Carotid Artery Stenosis

**DOI:** 10.3390/biomedicines11030881

**Published:** 2023-03-13

**Authors:** Ioan Alexandru Balmos, Emőke Horváth, Klara Brinzaniuc, Adrian Vasile Muresan, Peter Olah, Gyopár Beáta Molnár, Előd Ernő Nagy

**Affiliations:** 1 Doctoral School of Medicine and Pharmacy, I.O.S.U.D., George Emil Palade University of Medicine, Pharmacy, Science, and Technology of Targu Mures, 540142 Targu Mures, Romania; 2 Department of Anatomy, George Emil Palade University of Medicine, Pharmacy, Science, and Technology of Targu Mures, 540142 Targu Mures, Romania; 3 Vascular Surgery Clinic, County Emergency Clinical Hospital of Targu Mures, 540136 Targu Mures, Romania; 4 Department of Pathology, Faculty of Medicine, George Emil Palade University of Medicine, Pharmacy, Science, and Technology of Targu Mures, 38 Gheorghe Marinescu Street, 540142 Targu Mures, Romania; 5 Pathology Service, County Emergency Clinical Hospital of Targu Mures, 50 Gheorghe Marinescu Street, 540136 Targu Mures, Romania; 6 M3 Department of Surgery, George Emil Palade University of Medicine, Pharmacy, Science, and Technology of Targu Mures, 540142 Targu Mures, Romania; 7 Department of Medical Informatics and Biostatistics, George Emil Palade University of Medicine, Pharmacy, Science, and Technology of Targu Mures, 540139 Targu Mures, Romania; 8 Department of Biochemistry and Environmental Chemistry, George Emil Palade University of Medicine, Pharmacy, Sciences and Technology of Targu Mures, 540142 Targu Mures, Romania; 9 Laboratory of Medical Analysis, Clinical County Hospital Mures, 540394 Targu Mures, Romania

**Keywords:** calcification, inflammation, osteopontin, carotid stenosis, carotid endarterectomy

## Abstract

Background: severe carotid artery stenosis is a major cause of ischemic stroke and consequent neurological deficits. The most important steps of atherosclerotic plaque development, leading to carotid stenosis, are well-known; however, their exact timeline and intricate causal relationships need to be more characterized. Methods: in a cohort of 119 patients, who underwent carotid endarterectomy, we studied the histological correlations between arterial calcification patterns and localization, the presence of the inflammatory infiltrate and osteopontin expression, with ulceration, thrombosis, and intra-plaque hemorrhage, as direct signs of vulnerability. Results: in patients with an inflammatory infiltrate, aphasia was more prevalent, and microcalcification, superficial calcification, and high-grade osteopontin expression were characteristic. Higher osteopontin expression was also correlated with the presence of a lipid core. Inflammation and microcalcification were significantly associated with plaque ulceration in logistic regression models; furthermore, ulceration and the inflammatory infiltrate were significant determinants of atherothrombosis. Conclusion: our results bring histological evidence for the critically important role of microcalcification and inflammatory cell invasion in the formation and destabilization of advanced carotid plaques. In addition, as a calcification organizer, high-grade osteopontin expression is associated with ulceration, the presence of a large lipid core, and may also have an intrinsic role in plaque progression.

## 1. Introduction

Ischemic vascular brain disease, manifesting as brain infarction and white matter lesions (WMLs), is highly prevalent in the elderly, being the second leading cause of death globally, and can lead to permanent disability due to irreversible neurological and cognitive deficits [1]. It is most commonly caused by severe carotid stenosis or embolization from a high-risk carotid plaque resulting in a stroke. Atherosclerotic plaque development is driven by systemic and local factors that ultimately determine where plaques form and how they progress. Local susceptibility to plaque formation depends on the arterial microenvironment, including arterial mechanics, matrix remodeling, and lipid deposition through the regulation of vascular cell function [2].

The pathophysiology of atherosclerosis is well documented, considered a chronic, insidious lipid-driven inflammatory process with several potential contributors, which lead to ischemia of target tissues through narrowing of the large- and medium-sized arteries’ lumen. This process is initiated by endothelial dysfunction, followed by a cascade of cellular, functional, and molecular events, which imply the activation of inflammatory pathways [3]. Lipid deposition in the arterial wall causes an influx of macrophages to remove the lipid deposits, but the continuous increase in plasma cholesterol and the ineffective removal result in extracellular lipid accumulations, along with macrophage necrosis, leading to the formation of a necrotic core. This intimal inflammatory response activates smooth muscle cells in the underlying medial layer, which shift their phenotype and migrate into the neointima. Through fibroproliferative remodeling, smooth muscle cells contribute to the growing plaque size and lumen occlusion. However, this fibroproliferative response also forms a protective fibrous cap, rich in smooth muscle cells and collagen, which ensures the mechanical stability of the plaque and prevents plaque rupture [3].

Before the era of the inflammatory theory, vascular calcification was thought to be a passive process and a late manifestation of atherosclerosis. In the coronaries, calcification is a predictor of future cardiovascular events [4]; less is known about its causal relationships in the case of carotids. Recent histological evidence from human cerebral artery specimens has demonstrated that calcification may affect two different layers of the wall: the intima and/or the media. Calcification of the media is often associated with diabetes and chronic kidney disease. Intimal calcification is a typical feature of advanced atherosclerosis and manifests as two sub-types: micro- or spotty, early-stage calcification or macro-, sheet-like, and late-stage calcification [5]. Evidence from histopathological analyses and clinical imaging studies has confirmed that intimal calcification is more associated with plaque vulnerability. In contrast, medial calcification, particularly of the inner elastic lamina, contributes to artery stiffness rather than lumen stenosis [6].

Microcalcification develops due to activated macrophages and smooth muscle cells, which first form matrix vesicles and then crystallizing mineral deposits [4]. These can be identified by PET-CT imaging or optical coherence tomography; functionally, they increase the mechanical stress between the fibrous cap and lipid core, resulting in plaque rupture [7]. Several analyses have associated the late-stage “macrocalcification” with the presence of more differentiated smooth muscle cells and a more organized extracellular matrix, conferring plaque stability [8,9]; however, other recent studies have suggested that superficial, multiple calcifications and ulceration are associated with intra-plaque hemorrhage, and may represent higher-risk lesions [10]. Conversely, intraplaque hemorrhage and erythrocyte extravasation may stimulate osteoblastic differentiation and intralesional calcium phosphate deposition [11]. Osteopontin (OPN), a phosphoglycoprotein expressed in many tissues, consistently co-localizes with ectopic calcification. The molecule is a pro-inflammatory cytokine and can be further induced by reactive oxygen species, interleukin-1β (IL-1β), and tumor necrosis factor α (TNFα) [12]. Due to the density of glutamic and aspartic acid residues, OPN can fix a significant amount of calcium [13]. It was suggested that acute increases in OPN might have a protective role because it reduce calcification and assist in wound healing and neovascularization. However, its sustained elevation confers a high cardiovascular risk [12]. According to these data, the risk of a cerebrovascular event due to a vulnerable atheromatous plaque depends on the severity of vascular stenosis and plaque morphology and composition.

Taking these considerations, we studied the relationships between calcification patterns, the presence of inflammatory cell infiltrates, and histological signs of vulnerability: ulceration and thrombosis on arterial specimens of a cohort of patients who underwent carotid endarterectomy due to severe atherosclerosis.

## 2. Materials and Methods

### 2.1. Patients and Tissue Fragments

Carotid plaque specimens were collected by carotid endarterectomy from patients diagnosed with symptomatic carotid artery stenosis hospitalized between January 2020 and December 2022 in the Vascular Surgery Clinic—County Emergency Clinical Hospital and the Cardiovascular Surgery Clinic—Cardiovascular Disease and Transplantation Emergency Institute of Târgu Mureș (Romania).

**Figure 1:**
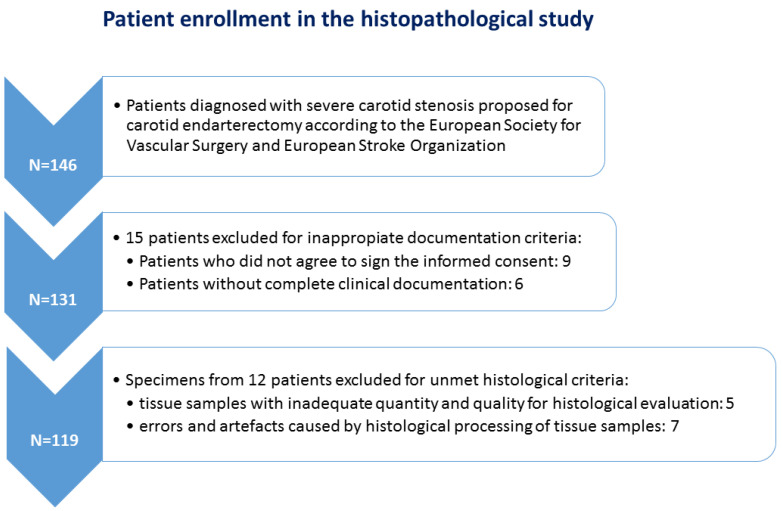
Flowchart of the study.

A total of 119 cases were selected for the histopathology study (plaques collected during carotid endarterectomy from 82 males and 37 females, all of them with severe carotid stenosis) based on strict criteria: patients with complete clinical documentation and the written informed consent of enrollment in the study, and last but not least, tissue samples with adequate quantity and quality for histological evaluation (Figure 1). Indication for carotid endarterectomy (CEA) was made on the clinician’s decision according to the European Society for Vascular Surgery and European Stroke Organization guidelines which recommend CEA for asymptomatic patients with carotid stenosis between 60–99% and for symptomatic patients with carotid stenosis between 50–99%. Prior to surgery, imaging tests (CT angiography and a Doppler ultrasonography) were performed to diagnose, localize, and grade the stenosis. The exclusion criteria consisted of patients with carotid near occlusion, which refers to severe carotid stenosis with distal vessel collapse and no significant improvement in stroke prevention within the first 5 years following endarterectomy [14,15], those who had experienced a major stroke, those with a second stenotic lesion in the intracranial segment of the internal carotid artery, and those who had previously undergone carotid endarterectomy on the same side [16]. Figure 1 shows the study flowchart with the main exclusion steps at the clinical and histopathological levels.

### 2.2. Patients Data Collection

Demographic information (age and sex) and clinical data (neurological symptoms at the time of admission, history of hypertension, diabetes mellitus, hypercholesterolemia, coronary heart disease, presence of atherosclerotic disease involving more than one vascular bed, current history of smoking, use of antiplatelet or anticoagulant, hypolipidemic, and anti-hypertensive drugs and previous stroke/transient ischemic attack history) were collected and checked against medical records. Hypertension, diabetes mellitus, and hypercholesterolemia have been defined according to recent guidelines [17,18,19]. A history of stroke was based on the definition in the World Health Organization criteria, including a syndrome of rapidly developing symptoms, with no apparent cause other than of vascular origin, of focal or global cerebral dysfunction lasting 24 h or longer, or leading to death [20]. From the laboratory examination results, we have noted recent data on absolute neutrophil and lymphocyte count and neutrophil/lymphocyte ratio. Our group’s epidemiological, clinical imagistic, and laboratory characteristics are summarized in Table 1, which also shows the data broken down to subgroups with and without an inflammatory cellular infiltrate.

This study was conducted according to the principles of the Helsinki Declaration. It was approved by the Ethical Committee of the George Emil Palade University of Medicine, Pharmacy, Science, and Technology of Targu Mures (no.906/2020) and the institutional review board of County Emergency Clinical Hospital of Targu Mures (no. 29496/2019) and of Cardiovascular Disease and Transplantation Emergency Institute of Târgu Mureș (no. 1680/2020). Written informed consent was obtained from each patient involved in this study.

### 2.3. Histological Processing

After surgery, the tissue samples were immediately fixed in 10% neutral buffered formalin and sent for histological analyses. Following decalcification in ethylene-diamine-tetra-acetic acid (EDTA) solution pH 7, all fragments were processed according to the standard methodology. For immunohistochemistry, consecutive histological sections were prepared. Morphological features of carotid plaques were examined in 4–5 µm sections stained with hematoxylin and eosin (H&E) by a senior pathologist (EH) blinded to the patient’s characteristics. Atherosclerotic plaques were classified accordingly to the Modified American Heart Association Classification in type IV-VII [21]. After establishing the grade, based solely on visual estimation of histological features without quantitative measurements, detection of characteristic signs of plaque vulnerability for each case was proposed, as follows: active mononuclear cells infiltration (macrophages and lymphocytes) within the atherosclerotic plaque, neovascularization within the lipid core, pattern of calcification (type, position, and extension), structure of lipid core (lipid-rich large necrotic core or hyaline rich core), intra-plaque hemorrhage, and fibrous cap damage (with or without parietal thrombus fragments) [22], each scored as being present or absent. Plaque rupture was identified by the presence of fibrous cap discontinuity with an endothelial defect of at least 1000 µm in width or a clear cavity formed inside the plaque [4], with or without thrombus, intra-plaque hemorrhage. The lipid core was categorized into large necrotic if cellular detritus predominated in its structure, along with macrophages with foamy cytoplasm and cholesterol crystals. In addition, we considered being fulfilled the criteria for an active mononuclear inflammatory infiltrate when macrophages and lymphocytes were observed around the core regardless of their quantity. The presence or absence of new vessels within the lipid core was also noted.

Focusing on their calcification type, plaques were included in four categories depending on the calcified patch distribution, size, and shape: (a) microcalcification (defined as a punctate pattern of numerous small micronodules/scattered small mineral foci), (b) nodular calcification (single/multiple stratified mineral deposits with a nodular aspect), (c) confluent/large calcification (a conglomerate of mineral material with irregular edges in collagen-rich plaque), and (d) osteoid metaplasia (mature bone with lamellar structure and bone marrow in a mineral mass of fibrocalcific plaque). Although there is no conventional standard of size, the consensus was taken into account that categorizes microcalcifications and macrocalcifications based on nodules of <50 and ≥50 μm, respectively [23]. Regarding position/location of calcification, we established two categories: superficial calcifications as calcified nodules located at the intimal–luminal interface or close to the lumen [10] and deep calcifications located in the thickness of the media or closer to the adventitia than to the lumen [4]. Extension of mineral mass was quantified depending on the occupied area from the total surface of the examined material and scored from 1 to 4, namely grade 1: less than 25%; grade 2: between 25–50%; grade 3: between 50–75% and grade 4: over 75%.

### 2.4. Investigation of the Osteopontin (OPN) Expression within the Atherosclerotic Plaque by Immunohistochemistry

Immunohistochemical staining was performed using Osteopontin (OPN) polyclonal antibody (pab73623) purchased from Covalab (Villeurbanne, France) in combination with EnVision FLEX/HRP (Agilent, Dako, Santa Clara, CA, USA) as secondary antibody and 3,3′-diaminobenzidine chromogen (DAB), respectively, according to the manufacturer’s instructions. Nuclei were counterstained by hematoxylin. For negative control, normal serum was substituted for the primary antibody. According to the extension of the brown color reaction product, plaques were categorized in low-grade (score 1), mild-grade (score 2), and high-grade (score 3) OPN expression. Score 1 was considered as few positive cells (<25 cells in no more than two areas of the plaque examined with 20× magnification). Score 2 was represented by immunolabelled cells in slightly increased numbers, not exceeding 50 elements in no more than two areas of the plaque examined with 20× magnification. When the number of positive cells exceeded 50 in number in the examined areas, samples were labeled as score 3.

### 2.5. Statistical Analysis

Categorical variables and transformed continuous variables were assessed for absolute and relative distribution frequency. Analysis of 2 × 2 or 3 × 2 contingency tables has been performed with the Fisher’s exact test and the Pearson χ^2^ test. Nonlinear logistic regression models were set for the prediction of ulceration and atherothrombosis. In all tests, *p*-values < 0.05 were considered statistically significant. Data processing was performed using Microsoft Excel 2016 (Microsoft Corporation, Redmond, WA, USA) and GraphPad Prism 9.5.0 (GraphPad Software LLC., San Diego, CA, USA).

## 3. Results

### 3.1. Study Group Characteristics

A total of 119, out of which 82 were male and 37 female patients, were enrolled in the study group, with a median age of 67 (61–72). All patients had severe carotid artery stenosis over 70% (mean ± SE 81.3 ± 0.7). A total of 84 individuals showed unilaterally, and 35 possessed bilateral involvement of the carotids. Seventeen patients suffered from various forms of sensory or motor aphasia, and hemiparesis/hemiplegia occurred in 13 study group members. A previous stroke history was confirmed in 76 subjects. The vast majority of cases were hypertensive (92.4%), and 27.7% were diabetic (type 1 and type 2). Eighty of them showed only carotid atherosclerosis; in 28 patients, two arterial bed involvement (carotids and limbs or coronary), and in 11 cases, three arterial bed involvement (carotids, limbs, and coronary arteries) was established. A total of 48.7% of the cohort were smokers, and except for four subjects, the rest presented hypercholesterolemia. No significant differences in the absolute neutrophil count, the absolute leucocyte count, and the neutrophil/lymphocyte ratio were observed (Table 1).

### 3.2. Histopathogical Features of Carotid Atherosclerotic Plaques

**Figure 2:**
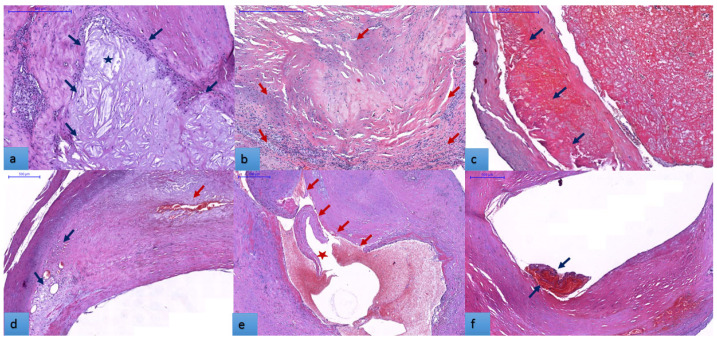
Representative sections of unstable carotid plaques from six different patients characterized by complex morphology and composition, H&E stain: (**a**). atherosclerotic plaque fragment with large necrotic lipid core (blue arrows; necrosis marked by star); (**b**). an abundant mononuclear inflammatory cell population (macrophages and lymphocytes) surrounds the lipid core and extends in the hyaline areas; (**c**). endarterectomy specimen shows erythrocyte aggregates in the plaque structure (intra- plaque hemorrhage) (blue arrows); (**d**). obvious intra-plaque neovascularization is seen both within and at the periphery of the plaque. The size of the neo-formed vessels varies between foci, some exceeding 10 µm (red arrow). (**e**). atheromatous plaque leading to narrowing and deformation of the lumen (blue arrows) shows fibrous cap discontinuity with an endothelial defect of at least 1000 µm in width (red arrows); (**f**). plaque ulceration in association with parietal thrombus represents a frequent cause of ischemic events. Bar represents 500 µm in all of the tissue sections.

First, we investigated the plaque architecture and histological features by light microscopy. These plaques, some unstable, others advanced, showed a varied histological composition. Characteristic signs of plaque vulnerability for each case were reported. A lipid-rich large necrotic core (Figure 2a) was detected in 58.8% of specimens. An active mononuclear cell component (macrophages and lymphocytes) was found in 63.02% of cases surrounding the necrotic core or extending to the hyalinised zones (Figure 2b). Accumulation of erythrocyte aggregates in the plaque structure (intra-plaque hemorrhage) (Figure 2c) was present in 32.8% of cases. Many plaques showed neovascularization by the proliferation of small, thin-walled microvessels with a collapsed lumen (66.4%), in most cases coexisting with inflammation and intra-plaque hemorrhage (Figure 2d). Ulcerated plaques with irregular and discontinuous fibrous caps (43.7%) led to thrombus formation in 16 cases (Figure 2e,f).

**Figure 3:**
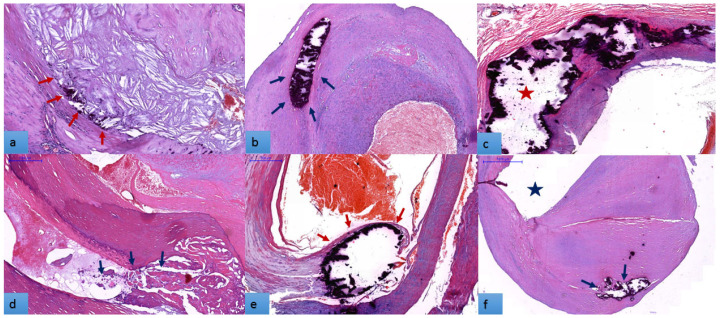
Calcification pattern in carotid plaques, H&E stain: Decalcified specimens from different cases show a various aspects and extension of mineral mass. (**a**). Large lipid core with sheet-like microcalcification forming a calcification front within fibrosis. (red arrows). Mononuclear inflammatory infiltrate is more abundant in the area around calcified foci; (**b**). nodular calcification with smooth rounded edges in the thickness of the fibrohyaline plaque (blue arrows); (**c**). plaque with heterogeneous structure, largely occupied by extensive calcification formed by conglomerated mineral nodules with irregular margins, extending towards the luminal surface of the plaque (red star); (**d**). plaque with large hyalinized areas in which osteoid metaplasia (trabecular bone and bone marrow foci) is also observed (blue arrows); (**e**). calcified nodule located at the intimal–luminal interface (red arrows), covered by thin fibrous cap (superficial calcification); (**f**). fibrous plaque with deep calcifications located in the thickness of the media (blue arrows, vascular lumen marked by star). Bar represents 500 µm in all of the tissue sections.

Because all examined plaques showed signs of calcification, the next step was to characterize the pattern, extent, and location of calcification. Regarding the intra-plaque calcification patterns, microcalcification, as numerous micronodules/scattered small mineral foci forming a calcification front within fibrosis (Figure 3a), was present by itself or in predominance in association with other types of calcification in 45.4% of cases. Seventy-one plaques (59.7%) showed a predominance of nodular calcification, as well-shaped single/multiple stratified mineral deposits with a nodular aspect (Figure 3b). Extensive (confluent) calcification (a conglomerate of mineral material with irregular edges (Figure 3c) dominated 34.5% of cases, and osteoid metaplasia (mature bone with lamellar structure and bone marrow (Figure 3d) was present in only 33 cases (27.7%). Depending on the location of the calcified foci in the thickness of the plaque, we observed a slight predominance in favor of superficial calcification over deep calcification 65 (54.6%) vs. 54 (45.4%). Mineral mass with grades 0–2 (54.6%) exceeded grades 3–4 (45.4%).

### 3.3. Osteopontin Expression

**Figure 4:**
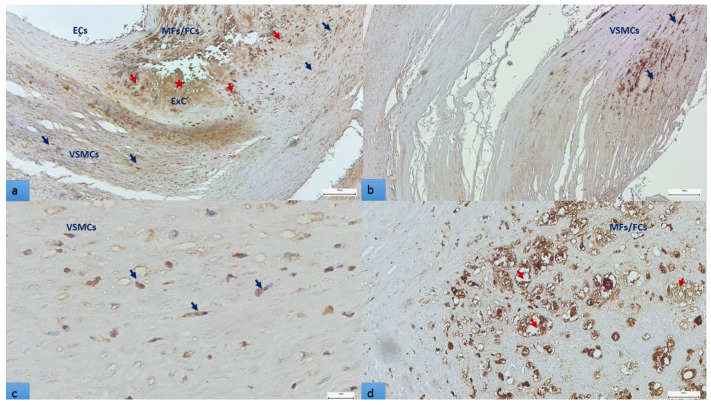
OPN-labelled cellular and extracellular structures in atherosclerotic plaque, immunohistochemical visualization (3,3′-diaminobenzidine chromogen): (**a**). OPN positive endothelial cells, macrophages (red arrows), fibroblasts (blue arrows). *OPN is also present extracellularly in the lipid core (star)*; bar represents 200 µm. (**b**,**c**). vascular smooth muscle cells are also OPN positive (blue arrows); bar represent 100 µm in (**b**), respectively, 20 µm in (**c**,**d**). OPN containing macrophages in the plaque at higher magnification. Many of these cells are foam cells with lipid droplets removed by organic solvents used for tissue processing, empty for OPN (red arrows); bar represents 50 µm.

After the characterization of carotid atherosclerotic plaque morphology, the ensuing investigation has focused on the role of OPN in plaque instability. We examined by immunohistochemical technique whether OPN expression is only cell-associated or is also present at the extracellular level in the plaque. In Figure 4, endothelial cells of the intima and neoformed vessels, macrophages, those transformed into foam cells, and fibroblasts are intensively cytoplasmic stained for OPN (Figure 4a). Vascular smooth muscle cells, other significant constituents of the atherosclerotic plaque and capable of transformation into foam cells, were also OPN positive (Figure 4b–d). In plaque structure without addition to the cells mentioned above, OPN staining of an extracellular component can also be observed. Details are shown on Figure 4.

This result indicates that extracellular OPN is an active component of atherosclerotic plaque, but to clarify if it interacts with proteins involved in the plaque structure requires further immunohistochemical studies. Because the immunohistochemical results showed that osteopontin is produced by multiple cells, we hypothesized that the amount of OPN in situ influences plaque instability. Based on the scoring system applied to quantify the OPN expression, we found that a low-grade OPN expression (score 1) is significantly associated with the absence of an inflammatory infiltrate (63.1% vs. 36.9%). In contrast, in those with mid-grade (score 2) and high-grade (score 3) OPN expression, the incidence of the infiltrate was 80.5% and 96.1%, respectively (*p* < 0.001). The low-grade OPN expression was present in 59% of plaques without a lipid core, but the high-grade OPN was characteristic for 84.6% of plaques with a lipid core (*p* < 0.001). The OPN score 3 was also significantly correlated with plaque ulceration (53.8%), whereas the OPN score 1 case in 70.2% showed no ulcers (*p* = 0.021). Moreover, in patients with polyvascular atherosclerotic disease affecting 3 arterial beds, 10 had an OPN score of 1, and only 1 had an OPN score of 3. In cases with one arterial bed involvement, the expression of OPN was more equilibrated (41.2% OPN score one vs. 23.7%) (*p* = 0.037). No relationship was observed with age, gender, neovascularization, thrombosis, intra-plaque hemorrhage, hypertension, aphasia, hemiparesis/hemiplegia, or bilateral carotid involvement. Microcalcification strongly correlated with osteopontin expression: its absence was observed in 73.7% of the cases with an OPN score of 1. On the contrary, its presence was documented in 80.7% of those with an OPN score of 3 (*p* < 0.001). Out of 65 cases with calcification of the superficial vessel wall layers, 22 had an OPN score of 1, and 19 had an OPN score of 3. However, in those with deep layer (media and adventitia) calcification, the distribution was unequal: 35 cases showed low, whereas only 7 cases showed high OPN expression (*p* = 0.003).

The extent of calcification, the nodular, the extended/confluent patterns, metaplasia, and the cumulated calcification pattern did not have significant associations. The distribution of OPN expression scores is indicated in Supplementary Table S1.

### 3.4. Comparison of the Inflammatory Infiltrate Positive (INF^+^) and Negative (INF^−^) Groups

Seventy-five patients possessed atheromatous plaques with a significant inflammatory infiltrate (INF), whereas 44 showed no elements of inflammation. Table 1 synthesizes the distribution of different variables in these two groups. The demographic parameters were similar in the two groups. The occurrence of aphasia was significantly lower in the inflammatory infiltrate negative (INF^−^) subgroup: 4.5% compared to 20% in the positive (INF^+^) subgroup (*p* = 0.027). In addition, the frequency of hemiparesis/hemiplegia was lower (4.5% vs. 14.7%) but without significance. The grade of carotid stenosis showed a borderline difference between the groups (*p* = 0.06). There were no essential differences concerning the incidence of diabetes, hypertension, bilateral carotid involvement, or previous stroke history. Polyvascular atherosclerotic disease with three arterial beds involvement was represented in 20.5% of cases in the INF^−^ group vs. 2.7% in the INF+ group, whereas solitaire carotid artery involvement equaled 50% in the INF^−^ and 77.3% in the INF^+^ group (*p* < 0.001).

### 3.5. The Distribution of Atheroma Calcification Patterns

Fifty-four plaques presented extended calcification (grades 3 and 4), out of which 30 (40%) were classified in the INF^+^ and 24 (54.5%) in the INF^−^ group. In addition, 54 specimens showed microcalcification from the overall group, with a significantly biased distribution between the groups: 42 (56%) in the INF^+^ group and only 12 (27.2%) in the INF^−^. Regarding the localization, the calcification was superficial in 54 and affected the deep layers in 65 cases. Superficial calcification characterized more (64%) INF^+^ plaques and less (38.4%) of those INF^−^ (*p* = 0.008). Among the calcification patterns, the nodular pattern was more frequent in the overall group than in the extended/confluent form (59.7% vs. 34.5%). The distribution of these patterns was significantly biased in the latter case: 26.7% of the INF^+^ group had extended/confluent calcification, compared to 47.4% of the INF^−^ cases (*p* = 0.027). Osteoid metaplasia was detected at 33 plaques in the entire group, being more frequent in those INF^−^ (40.9% vs. 22.6%), but this difference did not reach the significance threshold.

### 3.6. Treatment Correlations

Anti-hypertensive medication has been administered to all hypertensive patients (*n* = 110). A small subgroup was treated with anticoagulants (*n* = 7) preoperative (p.e.), and the majority of subjects received postoperative (p.o.) anti-coagulants (*n* = 95). Lipid-lowering treatment was administered to all, with the exception of three cases.

### 3.7. Correlations of Calcification Extent, Localization and Patterns with Ulceration, Thrombosis and Hemorrhagic Rupture of the Plaque

Ulceration occurred in a significantly higher proportion of plaques showing microcalcification: 34 of 54 vs. 18 of 65 (*p* < 0.001). Ulceration was relatively more frequent in those with superficial layer calcification than in plaques with deep layer involvement (*p* = 0.064). The nodular, the extended/confluent patterns, and the presence of metaplasia were not significantly associated with the ulcerative complication, neither as solitaire correlates nor in cumulative patterns (we classified in this group the occurrence of co-existing patterns, each component with a minimum weight of approximately 25%).

The presence of thrombosis was not correlated with calcification extent, localization, or patterns, as seen in Table 2.

The hemorrhagic rupture had a higher incidence in plaques with superficial calcification (27 of 65) than in those with deep layer calcification (12 of 54, *p* = 0.031). Further, the extended/confluent pattern was significantly associated with the lack of hemorrhagia (*p* = 0.019). No other calcification parameters or cumulative patterns correlated with the hemorrhagic rupture of the atheroma (Table 2).

### 3.8. Predictors of Plaque Ulceration

Univariate regression analysis revealed significant associations between plaque ulceration, the presence of single arterial bed involvement (*p* = 0.042), and morphological characteristics of the plaque: the lipid core (*p* = 0.004), micro-calcification (*p* < 0.001), and the presence of inflammatory infiltrate (*p* < 0.001). No significant relationships could be observed with diabetes, hypertension, a positive stroke history, unilateral vs. bilateral carotid involvement, age, gender, smoking, or hypercholesterolemia. A tendency for a lower odds ratio was registered in the presence of extended/confluent calcification pattern and in the highest vs. the lowest quartile of stenosis grade (*p* = 0.079 and *p* = 0.091). No other calcification patterns were correlated with ulceration (Table 3).

We constructed non-linear multiple logistic regression models to predict the ulceration of the plaques. In a model adjusted for the absence of polyvascular disease, a greater stenosis extent, hypertension, the presence of superficial and an extended/confluent pattern of calcification, and the presence of a lipid core, microcalcification (*p* = 0.003), and the presence of an inflammatory infiltrate (*p* = 0.007) remained significant predictors (Table 4). However, when we also adjusted for osteopontin expression, this abolished the significant influence of microcalcification and lipid core.

### 3.9. Predictors of Atherothrombosis

In univariate regression models, ulceration proved to be a strong predictor of thrombosis (*p* < 0.0001). The inflammatory infiltrates were also significantly associated (*p* = 0.046), whereas revascularization and the lipid core showed a borderline significance (*p* = 0.074 and *p* = 0.064, respectively). The other factors did not correlate, as shown in Table 5.

In univariate regression analysis, ulceration was strongly associated with plaque thrombosis (*p* < 0.0001). Further, the presence of inflammatory infiltrate was also significantly more frequent in those with thrombosis, (*p* = 0.046), whereas revascularization (*p* = 0.074) and the presence of the lipid core (*p* = 0.064) showed a tendency to significance. Age, gender, diabetes, hypertension, and smoking could not be associated, and no relationship has been revealed with the presence of polyvascular disease, unilateral involvement, and the grade of vascular stenosis. Furthermore, none of the calcification patterns could be correlated to the atherothrombotic complication (Table 6). In a multiple logistic regression model, ulceration and inflammation remained significant predictors, when adjusted for revascularization and the presence of the large lipid core (*p* = 0.002 and *p* = 0.007).

## 4. Discussion

Carotid endarterectomy and carotid stenting are two treatment modalities for patients with severe carotid stenosis. Histopathological processing of endarterectomy specimens provides useful information, which, together with clinical data and imaging investigation, contributes to the efficient secondary prevention of cerebrovascular events.

Carotid plaques have a complex morphology and composition, consisting of both extracellular (necrotic core, lipids, extracellular matrix proteins, lipids, and free cholesterol recognized as clefts) and cellular components dominated by inflammatory cells, smooth muscle cells, and fibrous tissue, which explains the existence of considerable differences in vulnerability between plaques with identical degrees of stenosis. In this context, histopathological assessment of plaque characteristics is one of the “gold standards” used to classify plaques as stable or unstable, first proposed by Lovett et al. in 2004 [22,24].

Although the precise sequence of lesion progression leading to plaque vulnerability is poorly elucidated, the morphological signs of instability as a potential indicator of stroke risk are well known. This hallmark includes a large lipid core, macrophage-mediated inflammatory changes, intraplaque neoangiogenesis/hemorrhage, ulceration, and microcalcification [25]. In this context, histological identification of these signs in endarterectomy specimens may provide valuable data concerning underlying plaque morphologies and should guide the treatment strategy to prevent further cerebral events.

Arterial plaques contain macrophages (MFs) involved in all steps of atherosclerosis. They are influenced by several cytokines and chemokines in the vessel wall and interact with local microenvironmental factors, which drive their differentiation into variable functional phenotypes. MFs can take up oxidized LDL and low-density lipoprotein (LDL)-derived cholesterol as lipid droplets and transform them into foam cells. These are significant players in the initiation and progression of atherosclerosis. The debris of macrophage-derived foam cells provides the major source of the necrotic core in the atherosclerotic plaque [26]. The liponecrotic tissue in atheroma with necrotic core appears to be developed by the structural collapse of the lipid core of atheroma due to the loss of elastic and collagen fibers; MFs are organically involved in this mechanism [27]. Matrix metalloproteinases synthesized by infiltrating macrophages are mainly responsible for their intra-plaque elastolytic and collagenolytic activity. Genetic deficiency of MMP-1a strongly suppresses atherogenesis in the aorta of apo E −/− mice [28]. Interestingly, collagen structure, along with synthesis, degradation, and remodeling of vascular wall elastin, proteoglycans, and glycosaminoglycans, modulate the phenotype of infiltrating inflammatory, but also of the resident cells [29].

In our case, histopathological examination of 119 endarterectomy specimens revealed the presence of a lipid-rich, large necrotic core (Figure 1a) in 58.8% of specimens. This core was associated with an active mononuclear cell component (macrophages and lymphocytes) that surrounded the necrotic core in 63% of cases. In patients without a high inflammatory component in their carotid plaques, the occurrence of cerebrovascular events such as aphasia, hemiparesis, or hemiplegia was significantly lower compared to the INF-positive subgroup. These results suggest that MF-mediated inflammation, initiated by the lipid content, destabilizes atherosclerotic plaques through the degradation of their cross-linked structural proteins. Increases in the size of the necrotic core may happen as a consequence of two factors, MF death, and impaired efferocytosis. In addition, necrosis contributes to forming an inflammatory microenvironment, enhanced oxidative stress, and thrombogenicity [3].

Fissuration and ulceration, landmarks of destabilization, are also the result of MF-released enzymatic activity. Tomas et al., in 159 carotid specimens obtained by endarterectomy, identified an altered metabolomic signature of unstable carotid plaques, comprising increased glycolysis and amino acid utilization along with low fatty-acid oxidation. A series of pro-inflammatory cytokines, like interleukins IL-1b, IL-6, IL-15, IL-17, and IL-18, are abundantly expressed in homogenates of the cluster characterized by the signature mentioned above [25,30]. Chemokines synthesized by MFs, like monocyte chemoattractant protein-1 (MCP-1) and MF inflammatory protein-1b (MIP-1b) were also up-regulated in unstable vs. stable plaques [30].

Our study defines ulceration, thrombosis, and intra-plaque hemorrhage as major morphological manifestations of atherosclerotic plaque vulnerability. Clinical findings support this assumption: the presence of ulceration, as the sole sign, is predictive of neurological symptoms and, together with advanced-grade stenosis, represents a high risk for stroke [31]. Sixty-one subjects were investigated by multi-detector computed tomography, and in 16 ulcerated plaques, no correlation was observed with the plaque volume. In contrast, ulcerative lesions were strongly associated with the presence of a lipid-rich content [31].

In univariate logistic regression models, we observed that the sizeable necrotic lipid core, but not hypercholesterolemia, was significantly associated with ulceration. However, this association was abolished in a multiple logistic regression model focused on ulceration, inflammation, revascularization and the lipid core. Regarding the infiltrating inflammatory elements of the atherosclerotic plaques and the circulating neutrophil count, or neutrophil/lymphocyte ratio, no correlation was found, even though several studies report higher neutrophil counts along the presence of microemboli detected by transcranial Doppler ultrasound in symptomatic patients [32]. Instead, the presence of the inflammatory cellular infiltrate, consisting predominantly of MFs, and microcalcification proved to be the strongest predictors of ulceration, which remained significant after adjustments for the stenosis grade, the presence of the lipid core, and superficial layer calcification. Further, ulceration and the inflammatory infiltrate, but not calcification extent or pattern, were defined as significant determinants of atherothrombosis, another important sign of plaque vulnerability.

An MF-rich inflammatory infiltrate was present in almost 2/3 of cases, so we investigated its histological correlates and compared it in INF^+^ and INF^–^ groups. We showed that there were no essential differences between these groups in terms of the incidence of diabetes, hypertension, bilateral carotid involvement, or previous stroke history. In contrast, atheroma calcification patterns and location showed a significantly biased distribution of microcalcification and superficial calcification in favor of the INF^+^ group. Even though the effect of calcification is considered biphasic, from pro-inflammatory properties of “microcalcification” to anti-inflammatory properties of “macrocalcification,” in our study, the distribution of extended/confluent pattern was almost twice less frequent in the case of the INF^+^ group (See Table 1). This result may suggest that extensive calcification, even if not a direct predictor of plaque ulceration and thrombosis, is less associated with inflammation and intra-plaque hemorrhage and might be considered a sign of plaque stability.

As reported in the literature, the calcification of atheromatous plaque is a remarkable feature of advanced atherosclerosis. It is triggered by inflammation, emerges as microcalcification, and develops through a spectrum of events to macrocalcification, with the formation of bone-like structures within the plaque. The release of matrix vesicles from macrophages and the death of VSMC initiates the calcification process of the plaque. Other factors are also involved in the process, including reduced levels of mineralization inhibitors or increased osteogenic transdifferentiation (VSMC pericytes) [3]. In our study, a part of the plaques showed mixed calcification patterns, containing foci with both microcalcification and different types of macrocalcification. In this case, the dominant patterns were considered, and for macrocalcification, cumulative patterns also were investigated. Our results demonstrated the presence of ulceration and intra-plaque hemorrhage in a significantly higher proportion of plaques with microcalcification. Furthermore, they showed dominance in plaques with superficial layer calcification compared to those with deep layer damage. No significant influence of the macrocalcification patterns was observed; no other single calcification parameters or cumulative patterns correlated with the hemorrhagic rupture of the plaque (See Table 2).

The role of calcification in the development of plaque progression is a controversial topic in the literature. The promoting role of microcalcification-induced stress on thin fibrous caps has been demonstrated in plaque rupture both by a three-dimensional blood-vessel modeling [33] and by imaging, histological and morphometric analysis [4,34]. Imagistic studies revealed some interesting aspects of calcification linkage with arterial wall inflammation: higher calcium scores appear along multiple sites in arteries in aging [35]. Carotid artery calcification was investigated in 130 patients included in the dal-PLAQUE study with 18F-labeled fluorodeoxyglucose positron emission tomography and computed tomography at entry and six months. The study revealed a poor overlap between vascular inflammation and calcification on multiple arterial beds. However, those with some calcification content at baseline showed more calcification progression than patients without. These arterial segments with progressive calcification showed a high [18F] FDG-PET signal, which highlights the putative causal role of inflammation in the progression of calcified lesions [35]. Macrocalcification is easily detected and quantified (calcium scores as predictive value for cardiovascular incidence) using the CT scan method. By contrast, microcalcification-the early stage of plaque calcification, is observed only with Positron Emission Tomography (PET)/CT Imaging and Optical Coherence Tomography (diagnostic methods that are not used in daily practice) [36]. However, the CT analysis of calcium subtypes is limited by the resolution and blooming artifacts. In this context, the histopathological examination of the endarterectomy specimens provides helpful information to the clinician to develop a treatment strategy. Molecular imaging of macrophages highlighted enhanced proteolytic activity via matrix metalloproteinases MMP-2, MMP-9, and MMP-13 [37]. MMP-9 is up-regulated by osteopontin, cleaves collagen and elastin substrates in the extracellular matrix, and determines hydroxyapatite crystal deposition [38]. In experimental conditions, the cysteine protease-activatable NIRF-imaging revealed that vascular calcification evolves parallel with bone osteolysis and might be driven by shared inflammatory driver mechanisms [37].

Several cytokine-type soluble regulators were described, which interact with different steps of calcification. For example, osteoprotegerin is elevated in the serum of patients with polyvascular atherosclerotic disease [39,40] and, in animal models, inhibits arterial calcification without affecting the development of the number and volume of atherosclerotic lesions [41]. Another regulator is osteopontin (OPN), a pro-inflammatory glycophosphoprotein involved in bone morphogenesis, a multi-faceted regulator of biomineralization, calcification, and tissue remodeling. Five isoforms of OPN may be expressed due to alternative splicing, which is a possible reason why it shows a diverse linkage with cardiovascular events in the population. OPN increases dramatically in acute stroke, and it is presumed that its early role is protective, attenuates vascular calcification, and promotes post-ischemic neovascularization. Paradigmatically, in chronic inflammatory pathways involving the vessel wall, OPN is probably harmful. It may be expressed in infiltrating macrophages, differentiating myofibroblasts, and endothelial cells of the vessel wall [42]. Wolak T. et al., in a study focusing on carotid atherosclerosis in hypertensive patients, demonstrated that none of the OPN “family members” have anti-inflammatory properties. OPN-N terminal fragment is associated with increased plaque inflammation [43].

In our tissue sections, a rather diverse cell population was stained for OPN. Morphologically, most of the OPN+ cells were macrophage-derived foam cells, VSMCs, and endothelial cells. We found that in our cohort, low-grade OPN expression was significantly associated with the absence of an inflammatory cell infiltrate. Furthermore, microcalcification was significantly correlated with OPN expression. Higher OPN scores were associated with plaque ulceration and the presence of a lipid core, and lower expression was observed in plaques without ulceration. Additionally, low OPN scores were observed in patients with polyvascular disease with three arterial bed involvement. These data converge with those obtained by Strobescu–Ciobanu in a recent study: the authors described in 49 carotid specimens a significant association of higher OPN with ulceration and inflammation of the atherosclerotic lesions [44]. However, no significant association could be defined with calcification; this discrepancy might lie in the applied methodology as calcium content was determined by carotid arteries’ ultrasonography based on plaque echogenicity.

## 5. Limitations

The results from the histological processing of the 119 specimens characterize the CA plaques at a well-determined moment (the occurrence of neurological complications) without the possibility of following the disease progression.

We considered that the semi-quantitative evaluation of plaque complexity accurately characterizes the most important histological features of the plaque and may predict the behavior of the plaque at the time of examination. Although it has been based solely on visual estimation and might have been limited by subjectivity and variability between observers, we chose this method for the properties of the specimens; in contrast to autopsy and experimental study subjects, the fragmented aspect of the specimens does not allow the accurate evaluation of the components and their ratio by morphometric methods. It may have affected our sensitivity to detect relevant associations.

## 6. Conclusions

In a previous morphometric study, we demonstrated that femoral and carotid plaques show different morphology and the tendency for calcification, suggesting that the mechanism is site-specific and vessel wall structure dependent [45]. In the current study, we focused on the carotid plaque’s intimate structure, with a particular emphasis on inflammation, as an important correlate and histological signs of vulnerability: ulceration, atherothrombosis, and hemorrhage.

Our results highlight the following critical issues related to carotid atherosclerotic plaques linked to severe carotid stenosis: (1) considerable differences in vulnerability signs between plaques with identical degrees of stenosis are due to complex morphology and composition; (2) in patients with carotid plaques with low/mild inflammatory components, the occurrence of cerebrovascular events (aphasia, hemiparesis/hemiplegia) is significantly lower compared to those with high degrees of inflammation, suggesting the role of pro-inflammatory MFs in atheroma vulnerability; (3) the presence of the inflammatory infiltrate and of the large necrotic lipid core increase the probability of ulceration; (4) ulceration and intra-plaque hemorrhage have appeared in a significantly higher proportion of specimens with microcalcification and dominated in plaques with superficial layer calcification compared to those with deep layer damage; and (5) our data suggest a potential role for OPN in the development of vulnerability: expression of OPN significantly correlated with microcalcification, and at the same time, higher OPN scores were associated with plaque ulceration and the presence of a sizeable necrotic lipid core.

Based on our results, we hypothesize that the lesions described are characteristic of advanced stages of atherogenesis; clarifying how plaque composition changes from apparent early lesion formation to clinically significant vascular stenosis require further longitudinal, multidisciplinary studies.

## Figures and Tables

**Table 1:** Clinical variables of the 119 atheromatous plaques, with vs. without inflammatory infiltrate.

Variable	Median (Quartile Range)/Mean ± SE	INF^+^ Group (*n* = 75)	INF^−^ Group (*n* = 44)	*p* Values
*Demographic and lifestyle variables*				
Age (years)	67 (61–72)	67 (60–73)	67.5 (63–71)	0.412
Gender (f/m)	37 (31.1)/82 (68.9)	23 (30.7)/52 (69.3)	14 (31.8)/30 (68.2)	1.000
Smoking (yes/no)	58 (48.7)/61 (51.3)	40 (53.3)/35 (46.7)	18 (40.9)/26 (59.1)	0.254
*Disease characteristics and comorbidities*				
Grade of stenosis * (%)	81.3 ± 0.7	80.3 ± 0.9	83.1 ± 1.2	0.062
Carotid atherosclerosis, uni- vs. bilateral (u/b)	84 (70.6)/35 (29.4)	50 (66.7)/25 (33.3)	34 (77.3)/10 (22.7)	0.297
Stroke history (y/n)	76 (63.9)/43 (36.1)	51 (68)/24 (32)	25 (56.8)/19 (43.2)	0.240
Occurrence of aphasia (yes/no)	17 (14.3)/102 (85.7)	15 (20)/60 (80)	2 (4.5)/42 (95.5)	0.027
Occurrence of paresis/plegia (yes/no)	13 (10.9)/106 (89.1)	11 (14.7)/64 (85.3)	2 (4.5)/42 (95.5)	0.128
Hypertension (y/n)	110 (92.4)/9 (7.6)	67 (89.3)/8 (10.7)	43 (97.7)/1 (2.3)	0.151
Diabetes (y/n)	33 (27.7)/86 (72.3)	18 (24)/57 (76)	15 (34.1)/29 (65.9)	0.471
Polyvascular disease (1/2/3 arterial beds affected)	80 (67.2)/28 (23.5)/11 (9.3)	58 (77.3)/15 (20)/2 (2.7)	22 (50)/13 (29.5)/9(20.5)	0.001
*Plaque calcification*				
Calcification extent (grade 3–4/grade 0–2)	54 (45.4)/65 (54.6)	30 (40)/45 (60)	24 (54.5)/19 (45.5)	0.142
Superficial/deep calcification	65 (54.6)/54 (45.4)	48 (64)/27 (36)	17(38.6)/27 (61.4)	0.008
Microcalcification (yes/no)	54 (45.4)/65 (54.6)	42 (56)/33 (44)	12 (27.3)/32 (72.7)	0.004
Nodular calcification (yes/no)	71 (59.7)/48 (40.3)	49 (65.3)/26 (34.7)	22 (50)/22 (50)	0.122
Extended/confluent calcification (yes/no)	41 (34.5)/78 (65.5)	20 (26.7)/55 (73.3)	21 (47.4)/23 (52.3)	0.027
Metaplasia (yes/no)	33 (27.7)/86 (72.3)	17 (22.6)/58 (77.3)	16 (40.9)/28 (59.1)	0.138
OPN expression (grade 1/2/3)	57(47.9)/36 (30.3)/26(21.8)	21(28)/29(38.7)/25(33.3)	36(81.8)/7(15.9)/1(2.3)	<0.001
*Biological variables and medication*				
Hypercholesterolemia (yes/no)	115 (96.6)/4 (3.4)	74 (98.7)/1 (1.3)	41 (93.2)/3 (6.8)	0.142
Abs. neutrophil count (10^9^/L)	5.29 (4.05–6.66)	5.51 (4.04–6.8)	5.07 (4.05–6.38)	0.293
Abs. lymphocyte count (10^9^/L)	1.95 (1.56–2.53)	1.95 (1.64–2.47)	1.93 (1.45–2.64)	0.686
Neutrophil/Lymphocyte ratio	2.70 (1.91–3.62)	2.8 (1.95–3.62)	2.55 (1.65–3.75)	0.338
Anti-hypertensive drugs (y/n)	110 (92.4)/9 (7.6)	67 (89.3)/8 (10.7)	43 (97.7)/1 (2.3)	0.151
Anticoagulant pre.op.(y/n)	7 (5.8)/112 (94.2)	0 (0)/ 75 (17.1)	37 (89.2)/7 (10.8)	0.077
Anti-aggregants pre.op.(y/n)	111 (93.2)/8 (6.8)	71 (94.6)/4 (5.4)	40 (90.9)/4 (9.1)	0.465
Anticoagulant post.op. (y/n)	95 (79.8)/24 (20.2)	62 (82.6)/13 (17.4)	33 (75)/11 (25)	0.349
Anti-aggregants post.op. (y/n)	119 (100)/0	75 (100)/0	44 (100)/0	-
Hypolipidemics (y/n)	116 (97.4)/3 (2.6)	73 (97.3)/2 (2.7)	43 (97.7)/1 (2.3)	1.000

Values of variables with normal distribution (marked with asterisk) are represented by the mean ± SE, whereas values of variables with abnormal distribution are shown as median (quartiles). Binomial variables are represented as absolute numbers and percentages (in brackets). Comparison of variables with discrete values was performed by the Fisher’s exact test (2 × 2 groups) and the Pearson χ^2^ test (3 × 2 groups). For the numeric variables, groups were compared with the Mann–Whitney U test. The level of statistical significance has been set to *p* = 0.05.

**Table 2:** Occurence of ulceration, thrombosis and hemorrhage in subgroups with various calcification patterns.

	Ulceration				Thrombosis				Hemorrhage			
		No	Yes	*p*		No	Yes	*p*		No	Yes	*p*
Localization	superficial	32	33	0.064	superficial	57	8	0.789	superficial	38	27	0.031
	deep	36	18		deep	46	8		deep	42	12	
Microcalcification	No	47	18	<0.001	No	58	7	0.422	No	48	17	0.117
	Yes	20	34		Yes	45	9		Yes	32	22	
Nodular pattern	No	28	20	0.85	No	40	8	0.422	No	36	12	0.165
	Yes	39	32		Yes	63	8		Yes	44	27	
Extended/confluent pattern	No	40	38	0.173	No	67	11	1	No	47	31	0.019
	Yes	27	14		Yes	36	5		Yes	33	8	
Osteoid metaplasia	No	47	39	0.68	No	77	9	0.14	No	55	31	0.277
	Yes	20	13		Yes	26	7		Yes	25	8	
OPN expression	1+	40	17	0.022	1+	50	7	0.924	1+	42	15	0.299
	2+	16	20		2+	31	5		2+	21	15	
	3+	12	14		3+	22	4		3+	17	9	
Cumulative patterns: Nodular AND/OR extended/confluent AND/OR Osteoid metaplasia	No	47	39		No	76	10		No	55	31	
	Yes	21	12	0.493	Yes	27	6	0.47	Yes	25	8	0.46

Variables are represented as absolute numbers; comparisons were performed with the χ^2^ test. The level of statistical significance has been set to *p* = 0.05.

**Table 3:** Univariate logistic regression of variables associated with plaque ulceration.

Variables	Coefficient	SD	OR (95%CI)	*p*-Level
Age (Q4/Q1)	0.089	0.251	1.19 (0.45–3.18)	0.721
Gender (M/F)	0.182	0.408	0.83 (0.38–1.82)	0.656
Diabetes (Yes/No)	−0.044	0.227	0.82 (0.36–1.86)	0.846
Smoking (Yes/No)	−0.019	0.387	1.02 (0.49–2.11)	0.959
Hypertension (Yes/No)	−1.060	0.733	0.34 (0.08–1.45)	0.150
Carotid atherosclerosis, unilateral/bilateral (u/b)	−0.335	0.412	0.71 (0.32–1.60)	0.418
Stroke history (Yes/No)	−0.084	0.385	0.92 (0.43–1.90)	0.826
Polyvascular disease (≥2 a. beds/single bed)	−0.647	0.315	26.68 (1.52–468.19)	0.042
Stenosis grade (Q4/Q1)	−0.416	0.244	0.43 (0.16–1.14)	0.091
Hypercholesterolemia (Yes/No)	0.836	1.170	2.30 (0.23–22.85)	0.476
Revascularization (Yes/No)	−0.284	0.391	0.75 (0.35–1.62)	0.468
Calcification extent (High/Low)	−0.297	0.373	0.43 (0.15–1.21)	0.427
Nodular (Yes/No)	0.225	0.379	1.15 (0.55–2.41)	0.554
Extended/confluent (Yes/No)	−0.716	0.405	0.54 (0.25–1.19)	0.079
Osteoid metaplasia (Yes/No)	−0.197	0.417	0.78 (0.34–1.77)	0.637
Mixed calcification pattern (Yes/No)	−0.451	0.336	0.68 (0.30–1.57)	0.182
Microcalcification (Yes/No)	1.568	0.398	4.44 (2.04–9.63)	<0.001
Superficial/deep	−0.721	0.380	2.06 (0.98–4.35)	0.059
Lipid core (Yes/No)	1.190	0.402	3.28 (1.49–7.24)	0.004
Inflammatory infiltrate (INF^+^/INF^−^)	2.015	0.474	7.50 (2.96–19.00)	<0.001

**Table 4:** Summary of multiple logistic regression analysis of the factors correlated to atheromatous plaque ulceration in the overall patient group (*n* = 119).

Model 1. Variables	Estimate	SD	Odds Ratio (95% CI)	*p* Value
Microcalcification	1.470	0.491	4.44 (2.04–9.63)	0.003
Lipid core (Yes/No)	0.104	0.526	3.28 (1.49–7.24)	0.843
Superficial/deep	0.376	0.509	2.06 (0.98–4.35)	0.460
Extended/confluent calcification	−0.048	0.496	0.54 (0.25–1.19)	0.922
Inflammatory infiltrate (INF^+^/INF^−^)	1.575	0.574	7.50 (2.96–19.00)	0.007
Stenosis grade (Q4:Q1)	−0.376	0.300	0.43 (0.16–1.14)	0.212
Polyvascular disease (≥2 a. beds/single bed)	−0.390	0.409	26.68 (1.52–468.19)	0.342
Hypertension (Yes/No)	−0.042	0.880	0.34 (0.08–1.45)	0.961

**Table 5:** Univariate logistic regression of variables associated with thrombosis of the plaque.

Variables	Estimate	SD	OR (95%CI)	*p*-Level
Age (Q4/Q1)	0.150	0.364	1.60 (0.25–10.29)	0.681
Gender (M/F)	−1.284	0.408	1.44 (0.29–7.06)	0.656
Diabetes (Yes/No)	0.313	0.301	1.69 (0.56–5.09)	0.300
Smoking (Yes/No)	0.230	0.343	0.79 (0.27–2.29)	0.675
Hypertension (Yes/No)	0.233	1.095	1.26 (0.14–10.83)	0.821
Carotid atherosclerosis, unilateral/bilateral	−0.669	0.675	1.95 (0.52–7.33)	0.323
Stroke history (Yes/No)	0.603	0.612	1.82 (0.55–6.06)	0.326
Polyvascular disease (≥2 a. beds/single bed)	0.545	0.547	1.67 (0.57–4.88)	0.320
Stenosis grade (Q4/Q1)	−0.189	0.375	0.68 (0.15–2.98)	0.615
Revascularization (Yes/No)	1.409	0.783	4.09 (0.88–18.98)	0.074
Nodular (Yes/No)	−0.454	0.539	0.63 (0.22–1.82)	0.401
Extended/confluent (Yes/No)	−0.167	0.577	0.84 (0.27–2.62)	0.772
Osteoid metaplasia (Yes/No)	0.834	0.552	2.30 (0.78–6.80)	0.133
Mixed calcification pattern (Yes/No)	0.524	0.562	1.69 (0.56–5.09)	0.353
Microcalcification (Yes/No)	0.505	0.541	1.65 (0.57–4.79)	0.353
Superficial/deep	0.214	0.537	0.80 (0.28–2.31)	0.690
Lipid core (Yes/No)	1.252	0.670	3.49 (0.93–13.01)	0.064
Inflammatory infiltrate (INF^+^/INF^−^)	1.572	0.782	4.81 (1.04–22.33)	0.046
Ulceration (Yes/No)	23.78	-	61.02 (3.55–1046)	0.005

**Table 6:** Summary of multiple logistic regression analysis of the factors correlated to thrombotic complication in the overall patient group (*n* = 119). ***Model 2***.

Variables	Estimate	SD	Odds Ratio (95% CI)	*p* Value
Lipid core (Yes/No)	0.609	0.823	3.28 (1.49–7.24)	0.843
Inflammatory infiltrate (INF^+^/INF^−^)	0.623	0.971	7.50 (2.96–19.00)	0.007
Revascularization (Yes/No)	2.021	0.832	0.43 (0.16–1.14)	0.212
Ulceration (Yes/No)	3.335	1.082	61.02 (0.08–1.45)	0.002

## Data Availability

Data spreadsheets are available at https://data.mendeley.com/drafts/y6t5yxdsdd, doi: 10.17632/y6t5yxdsdd.2 (accessed on 9 February 2023).

## Supplementary Materials

The following supporting information can be downloaded at: https://www.mdpi.com/article/10.3390/biomedicines11030881/s1, Table S1: Associations of the OPN expression scores.
