# Supplementary material for: Inflammation, Microcalcification, and Increased Expression of Osteopontin Are Histological Hallmarks of Plaque Vulnerability in Patients with Advanced Carotid Artery Stenosis
Source: Biomedicines. 2023 Mar 13;11(3):881. doi: 10.3390/biomedicines11030881 (PMC10045225; doi:10.3390/biomedicines11030881)
Supplement: Supplementary file 1 [file biomedicines-11-00881-s001.zip › biomedicines-2243485-supplementary.pdf]

Supplementary Table S1. Associations of the OPN expression scores.

Variable	OPN expression			p values
	1+	2+	3+	
Demographic and lifestyle variables				
Age (Q3/Q1)	16/13	9/12	7/8	0.842
Gender (f/m)	14/43	15/21	8/18	0.221
Smoking (yes/no)	31/26	16/20	14/12	0.618
Disease characteristics and comorbidities				
Grade of stenosis (Q3/Q1)	19/13	6/11	6/6	0.275
Carotid atherosclerosis, uni- vs. bilateral (u/b)	43/14	24/12	17/9	0.534
Stroke history (y/n)	33/24	25/11	18/8	0.429
Occurrence of aphasia (yes/no)	8/49	6/30	3/23	0.847
Occurrence of paresis/plegia (yes/no)	5/52	4/32	4/22	0.668
Hypertension (y/n)	54/3	34/2	22/4	0.232
Diabetes (y/n)	18/39	11/25	4/22	0.458
Polyvascular disease (3 vs. 1 arterial bed affected)	10/33	1/28	0/19	0.037
Plaque calcification				
Extent of calcification (grade 3-4/grade 0-2)	29/28	15/21	10/16	0.497
Superficial/deep calcification	35/22	12/24	7/19	0.003
Microcalcification (yes/no)	15/42	18/18	21/5	<0.001
Spotty/ nodular calcification (yes/no)	32/25	24/12	15/11	0.585
Extended/ confluent calcification (yes/no)	23/34	10/26	8/18	0.418
Metaplasia (yes/no)	19/38	10/26	4/22	0.288
Biological variables of plaque vulnerability				
Inflammatory infiltrate (INF^+/INF^-)	21/36	29/7	25/1	0.142
Revascularization (yes/no)	38/19	26/10	15/11	0.488
Lipid core (yes/no)	23/34	25/11	22/4	<0.001
Ulceration (yes/no)	17/40	20/16	14/12	0.022
Thrombosis (yes/no)	7/50	5/31	4/22	0.924
Hemorrhage (yes/no)	15/42	15/21	9/17	0.299

Binomial variables are represented as absolute numbers. Comparison of variables with discrete values was performed by the Pearson $\chi2$ test (3x2 groups). The level of statistical significance has been set to p=0.05. Q1-quartile 1, Q3-quartile 3.
